# Unidirectional Nanopore Dehydration Induces an Anisotropic Polyvinyl Alcohol Hydrogel Membrane with Enhanced Mechanical Properties

**DOI:** 10.3390/gels8120803

**Published:** 2022-12-08

**Authors:** Feng-Ya Jing, Yu-Qing Zhang

**Affiliations:** School of Biology and Basic Medical Sciences, Soochow University, RM702-2303, No. 199, Renai Road, Dushuhu Higher Edu. Town, Suzhou 215123, China

**Keywords:** PVA, unidirectional nanopore dehydration, hydrogel, mechanical properties

## Abstract

As a biocompatible, degradable polymer material, polyvinyl alcohol (PVA) can have a wide range of applications in the biomedical field. PVA aqueous solutions at room temperature can be cast into very thin films with poor mechanical strength via water evaporation. Here, we describe a novel dehydration method, unidirectional nanopore dehydration (UND). The UND method was used to directly dehydrate a PVA aqueous solution to form a water-stable, anisotropic, and mechanically robust PVA hydrogel membrane (PVAHM), whose tensile strength, elongation at break, and swelling ratio reached values of up to ~2.95 MPa, ~350%, and ~350%, respectively. The film itself exhibited an oriented arrangement of porous network structures with an average pore size of ~1.0 μm. At 70 °C, the PVAHMs formed were even more mechanically robust, with a tensile strength and elongation at break of 10.5 MPa and 891%, almost 3.5 times and 2 times greater than the PVAHM prepared at 25 °C, respectively. The processing temperature affects the velocity at which the water molecules flow unidirectionally through the nanopores, and could, thus, alter the overall transformation of the PVA chains into a physically crosslinked 3D network. Therefore, the temperature setting during UND can control the mechanical properties of the hydrogel membrane to meet the requirements of various biomaterial applications. These results show that the UND can induce the ordered rearrangement of PVA molecular chains, forming a PVAHM with superior mechanical properties and exhibiting a greater number of stronger hydrogen bonds. Therefore, the novel dehydration mode not only induces the formation of a mechanically robust and anisotropic PVA hydrogel membrane with a porous network structure and an average pore size of ~1.0 μm, but also greatly enhances the mechanical properties by increasing the temperature. It may be applied for the processing of water-soluble polymers, including proteins, as novel functional materials.

## 1. Introduction

Polyvinyl alcohol (PVA) is a linear synthetic polymer obtained from the partial or complete hydrolysis of polyvinyl acetate, which removes the acetate group [1]. Due to the presence of a large number of hydroxyl groups in its molecular structure, PVA is highly hydrophilic and biocompatible [2,3]. It is easy to make porous hydrogels using PVA that have many advantages, such as non-toxicity, biodegradability, excellent mechanical properties, and a high degree of water absorption. The materials have, thus, been widely used in agriculture, forestry, medicine, chemicals used in daily life, environmental protection, and desert control. In particular, PVA has a large number of applications in the field of biomedical materials, with a significant number of studies having been conducted [4,5,6,7]. PVA hydrogels have a 3D-networked pore structure that has an affinity for proteins on its surface [8]; it can, thus, also be used as a vascular “shield” or adhesion barrier to prevent vascular adhesions at the surgical site, which reduces the incidence of complications [9,10,11]. PVA hydrogel materials also do not produce cytotoxic or mutagenic effects, nor do they show any signs of systemic toxicity and sensitization [12]. There is a significant amount of literature proving that PVA can be degraded by some microbial enzymes and is, thus, environmentally friendly [13,14,15,16], leading it to be widely used in commercial and industrial products [17,18].

PVA hydrogel preparation methods can be mainly divided into physical [19] and chemical crosslinking methods [20]. Chemical crosslinking involves crosslinking agents or chemical reactions initiated by radiation to form new intermolecular chemical bonds; in these methods, PVA tends to undergo hydroxyl aldol condensation reactions in the presence of crosslinking agents such as aldehydes [21]. Physical crosslinking is achieved without the addition of any chemicals and is accomplished mainly through the formation of hydrogen bonds between the hydroxyl groups of adjacent parts of the PVA molecule chain, resulting in the formation of locally ordered microcrystals that become crosslinking points [22]. Physical crosslinking generally utilizes pure PVA gels; the gelling process is simple and has relatively few toxicological safety issues [23].

Currently, the most commonly used physical crosslinking method for the preparation of PVA hydrogels is the cyclic freeze–thawing method [24,25], a typical method that improves the properties of materials by promoting the formation of hydrogen bonds, thereby increasing their crystallinity [26,27]. Generally, temperatures of −20 °C or below restrict the ability of polymer chains to form crystal nuclei, while thawing at 25 °C or above allows the crystals to grow. Thus, increasing the number of freeze–thaw cycles allows for the refinement of the crystal structure and the degree of crystallinity of the PVA film [28]. It has also been reported that the PVA films obtained after seven freeze–thawing cycles followed by annealing at 130 °C can show substantially improved tensile strength and Young’s modulus values [29]. These freeze–thaw methods form hydrogels with pore sizes that are too large for the immobilization of microorganisms and tissue cells and the slow release and delivery of enzymes and drugs. In addition, like most polymeric materials, the mechanical properties of the PVA are isotropic, unlike the soft tissues of most anisotropic organisms. Millon et al. [30] were able to induce directional mechanical properties into a hydrogel by applying a controlled strain to the PVA sample during low-temperature thermal cycling. The mechanical stability of PVA hydrogel materials has also been improved by freezing the container starting from the bottom up during multiple cycles of freeze–thawing, which promotes the production of ordered microcrystals [31]. All these methods allow for the preparation of a complex series of composites with a neatly arranged microporous structure [32,33]. This directional freeze–thawing results in anisotropic porous PVA hydrogels with high tensile strengths (0.3–1.2 MPa) and moderate compressive moduli (0.03–0.10 MPa). The anisotropic mechanical properties of these hydrogels can be attributed to the directional arrangement of the crystalline regions perpendicular to the freezing direction [34,35]. Oriented PVA hydrogels can also be obtained using enhanced compression processing, such as when producing implant materials for bone tissue engineering [36]. While these methods of directed freezing or subsequent stretching and compression processing are simple and produce PVA hydrogels with increased tensile strength and elastic moduli, there is still room for improvement with regards to the controllability of the process, the mechanical properties of the final materials, the immobilization of microorganisms and tissue cells, and the slow release of the enzymes and drugs.

In this paper, we report on a simple, green method of producing anisotropic PVA hydrogel membranes, which can be further processed into soft, transparent, porous networked structures that possess excellent mechanical properties and high swelling ratios through the use of UND technology.

## 2. Results

### 2.1. UND Preparation of PVAHM

**Figure 1:**
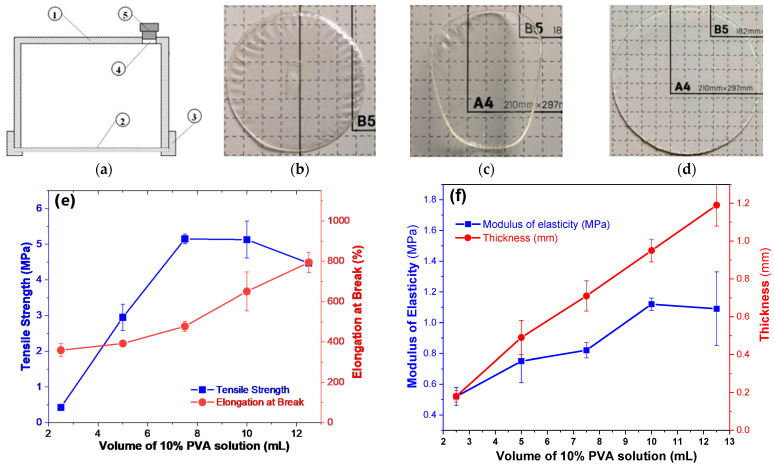
The schematic structure of the UND mold (**a**), the appearance of PVAHMs (**b**–**d**), and the effects of the PVA level on the mechanical properties and thickness of PVAHMs (**e**,**f**). (**a**) 1—Cup body; 2—nanopore filter membrane; 3—fixing flange ring; 4—hole of sample feeding; 5—rubber plug. (**b**–**d**) Images of a PVA membrane in semi-dry, dry, and swollen states in water at equilibrium (24 h); the size of the small squares in the background of figures is 10 mm × 10 mm. (**e**,**f**) The evolution of some of the mechanical properties of the PVAHM in the semi-dry state (30 mm × 5 mm rectangular strips) as a function of the initial volume of the PVA solution used for membrane preparation (*n* = 5).

PVA hydrogel membranes are produced when the PVA solution transitions from a liquid to a solid state; this occurs because the mold is designed in such a way that only the bottom nanopore film is permeable to water, resulting in the directionality of the PVA dehydration. In other words, the PVAHMs are formed via UND. Figure 1b shows the soft state of a hydrogel membrane that has a mass of 3.359 g just after it was removed from the mold; when completely dried, the membrane weighed 2.272 g (Figure 1c). After soaking the membrane in deionized water for 24 h and allowing it to reach its equilibrium swelling, the sample had a weight of 10.901 g (Figure 1d). Practically, the swelling ratio increased to about 500%. In fact, the dried PVAHM was found to be a very hard and tough material. In addition, the diameter of the membrane in its wet state significantly increased from ~70 mm in the dry state (Figure 1c) to ~105 mm in the swollen state (Figure 1d). The large value of the equilibrium swelling ratio of the PVAHM was equivalent enough to say that the PVAHMs prepared using this method can absorb a significant amount of water. 

### 2.2. Thickness

Five different volumes of 10% PVA solution were used to prepare five PVAHMs with different thicknesses (Figure 1e,f). The effect of the volume of the PVA solution used on the mechanical properties of the PVAHMs was analyzed. The maximum load (F_max_) of the PVAHMs increased as the thickness of the PVAHMs increased. The elongation at break (BE) also ascended with the volume of PVA solution initially used. Because of the precision of the stretching apparatus, the PVAHMs that resulted from volumes of less than 2.5 mL and more than 12.5 mL could not be measured accurately. However, within the measured range, it was observed that PVAHMs with greater thicknesses exhibited better mechanical properties expressed in increasing tensile strength, elongation at break, and modulus of elasticity values. 

### 2.3. PVA Molecular Weight

Due to the synthesis procedure (the radical polymerization of vinyl acetate followed by the hydrolysis of poly(vinyl acetate) to PVA), PVA is a polydisperse polymer characterized by an average molecular weight. To compare the effects of the different molecular weight values and viscosities of the PVA on the corresponding hydrogel films as formed via the UND method, PVAs of different molecular weights were purchased from the same brand (Aladdin, CAS No. 9002-89-5).

Table 1 presents the effect of the type of PVA on the formation of the respective UND-based PVAHMs and their properties. The six PVA samples shown in Table 1 could all form PVAHMs when prepared using the UND method. However, the films formed using PVA-203, PVA-210, and PVA-224 could not maintain their stability after being immersed in water and gradually dissolved. Although the PVA-105 film was stable in water, its tensile properties were poor; the sample could easily be broken with a gentle pull by hand, and its stress–strain curve could not be measured using laboratory instruments. The other two PVA samples, PVA-117 and PVA-124, had average molecular weights of 145,000 and 190,000 kDa, respectively; these formed PVAHMs that possessed good mechanical properties. These results show that whether the hydrogels formed using aqueous PVA solutions via UND possessed good mechanical properties that were primarily related to the degree of alcohol dissolution, followed by the molecular weight. For example, the molecular weight of PVA-105 was only 47,000 kDa: one-third and one-fourth the molecular weights of PVA-117 and PVA-124, respectively. It should be noted that PVA-117 is toxic and may cause damage to organs or can be fatal if it is swallowed and allowed to enter the respiratory tract, which comes from the Safety Data Sheet (SDS) for PVA-117. Therefore, PVA-124 was finally selected as the study material for this experiment.

### 2.4. Temperature Influence on Physicomechanical Properties

**Figure 2:**
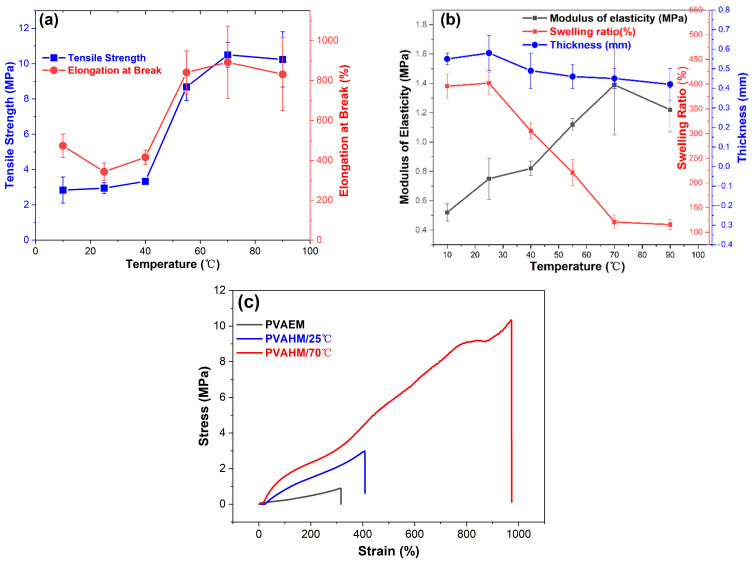
(**a**,**b**) The effects of temperature on the physicomechanical properties of PVAHMs (*n* = 5) and (**c**) the stress–strain curves of PVAEM prepared at 25 °C and PVAHMs prepared at 25 °C and 70 °C (the PVA hydrogel film was prepared from 5 mL of 10% PVA aqueous solution; strips of 30 mm × 5 mm in the dry state were used after rehydration as testing samples at 45% RH; average thickness of the strips ~0.55 mm).

The preparation of PVAHMs can also be influenced using different processing temperatures. The temperature affects the velocity at which the water molecules flow unidirectionally through the nanopores, and could, thus, alter the overall transformation of the PVA chains into a physically crosslinked three-dimensional network. From a physicomechanical point of view, the influence of the preparation temperature on the tensile strength, elongation at break, and modulus of elasticity of the tested PVAHMs seems to mirror a behavior specific to a rubber-like state or simply a rubbery state [37]. Thus, in such a state, the PVA chain segments between the crosslinking points (well-aligned microcrystalline regions) undergo a micro-Brownian motion with larger and larger intensity as the temperature increases [38]. Direct consequences of this behavior can be seen in samples shrinking in the absence of an external stress being applied and on the other hand in increasing values of the tensile strength, elongation at break, or modulus of elasticity with ascending temperatures. However, in this study, all of the physicomechanical measurements were performed at the same temperature (room temperature) on the PVAHMs prepared using UND methods at six different temperatures (PVAHM/10 °C, PVAHM/25 °C, PVAHM/40 °C, PVAHM/55 °C, PVAHM/70 °C, PVAHM/90 °C) and then rehydrated. This means that the crosslinked structures of PVAHMs possess some peculiarities that are strongly determined by the preparation temperature. Most likely, these have to do with the spatial expansion (size) of the crosslinking points (microcrystalline regions functioning as physical crosslinkages) and also the density of the crosslinking for the whole PVAHM network. As a consequence, based on the ascending tendencies, as almost linearly shown by the tensile strength, elongation at break, and modulus of elasticity of semi-dry PVAHMs prepared in the temperature range of 25–70 °C (as can be seen in Figure 2a,b), one can hypothesize that the general flexibility of the samples increases with the preparation temperature. In fact, the amorphous regions of PVA confer an increased flexibility to the PVAHMs, and conversely the microcrystalline regions of PVA restrain the degrees of freedom of movement of the well-aligned PVA chains, i.e., their micro-Brownian motion in the swollen state. In other words, at higher preparation temperatures (within 25–70 °C), the density of crosslinking could become gradually larger and the spatial expansion of the microcrystalline regions progressively smaller. As a result, the general flexibility of the PVA membranes in the swollen state may be high enough and the swelling capacity quite small (due to a high degree of crosslinking). On the contrary, at low temperature belonging to the same temperature range, the spatial expansion of the crosslinking points becomes larger and larger and the density of the crosslinking lower and lower. This is why the overall flexibility tends to reach its minimum value for the rehydrated samples and the equilibrium swelling its maximum value. This interplay between the spatial expansion and the density of crosslinking strongly influenced by temperature satisfactorily explains both the increases in tensile strength, elongation at break, and modulus of elasticity with the preparation temperature for the rehydrated PVA membranes on one hand and the descending tendency of the swelling ratios with the preparation temperature on the other hand (Figure 2a,b). At the highest preparation temperature employed here (90 °C), the physically crosslinked points and regions became looser and weaker and some of them simply no longer formed, while the tendencies discussed above followed a much more attenuated shape. Last but not least, the mutual influence of the spatial expansion–crosslinking degree is supported by the stress–strain dependences recorded at two different temperatures, as can be seen in Figure 2c. Thus, the samples prepared at 25 °C and then rehydrated displayed a stress–strain curve over a strain range of about 0–400% (low flexibility and crosslinking degree), while the same types of samples obtained at 70 °C (also rehydrated afterwards) exhibited a stress–strain trace evolved over a much wider range of relative elongation of about 0–900% (high flexibility and crosslinking degree). 

### 2.5. Stress and Strain Curves

Figure 2c presents the stress–strain curves of PVAHMs formed by directional nanopore dehydration at 25 °C and 70 °C (at a relative humidity of approximately 45%) and PVAEM as a control formed by water evaporation (0.89/2.97–312/407%). It can be seen that the tensile strength and elongation at break of this casted control sample were 0.9 MPa and 312%, respectively, while the tensile profiles of the UND-based PVAHM produced at 25 °C were relatively similar to the control. The tensile strength of the sample produced at 25 °C (PVAHM/25 °C) was close to 3.0 MPa and had a strain of almost 400%, whose tensile strength was more than 3 times higher than that of the control sample, while the elongation at break also increased by 30%. In contrast, when the temperature of the dehydration process was increased to 70 °C (PVAHM/70 °C), its tensile strength was almost 10.5 MPa, nearly three times the value of PVAHM/25 °C. In addition, its strain had more than doubled, approaching a value of 900%. These results indicate that temperature has a significant influence on the structural and mechanical properties of PVA hydrogel films. As a partial concluding remark, it could be said that the temperature can be used to modulate the relevant properties of PVA hydrogel films that are prepared to meet specific applications. 

### 2.6. Thermal Properties

**Figure 3:**
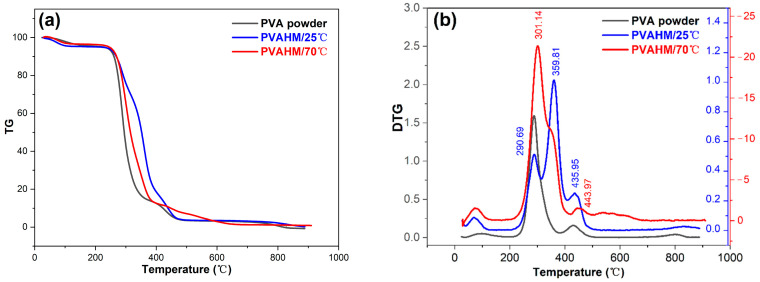

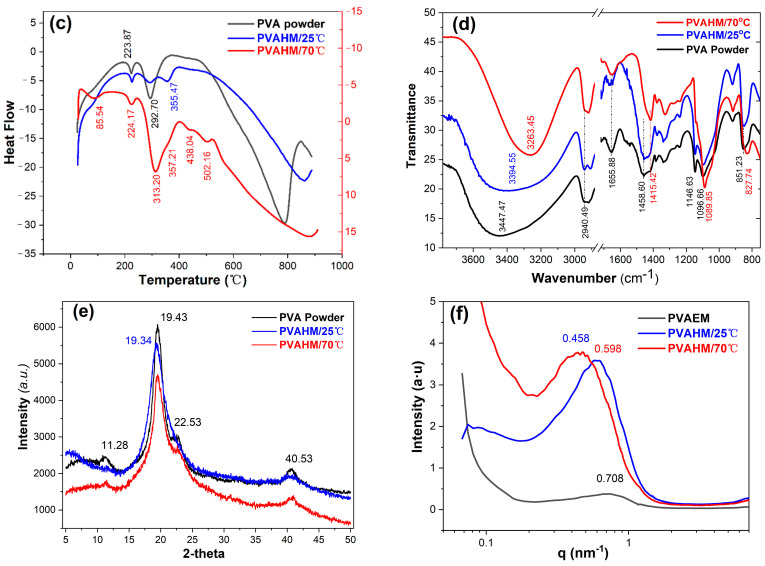
(**a**–**c**) TG, DTG, and DSC thermograms; (**d**) FTIR spectra; (**e**) XRD diffractograms of pure PVA powder, PVAHM/25 °C, and PVAHM/70 °C; and (**f**) SAXS patterns of PVAEM, PVAHM/25 °C, and PVAHM/70 °C.

The TG and DTG curves of pure PVA powder revealed the presence of three major weight loss regions; these were expressed as three distinct peaks in the DTG curve [39,40]. Figure 3a–c shows that the first region, located between 50 and 190 °C, is due to the evaporation of physically weakly bound water and chemically strongly bound water due to the relaxation of the crystalline domains in PVA; the weight loss experienced by the powder was approximately 4.56 wt.%. The second transition zone occurs between 230 and 370 °C; this is due to the degradation of the PVA polymer caused by the melting of the crystalline domains as hydrogen bonds begin to break. The total weight lost in this second stage is about 81.56%. The third stage peaks at 432.9 °C and is due to the breaking of the main chain of the PVA polymer, also known as carbonization; the total weight lost at this stage is only 10.75%. The total weight lost at 500 °C is 96.87%.

In contrast, four weight loss regions were observed during the thermal decomposition of PVAHM/25 °C (Figure 3a–c). The first region occurred at a temperature range of 50–190 °C, similar to that of pure PVA powder, with a weight loss of about 4.81 wt.%, due to relaxation in the crystalline domain of the PVA. Unlike the pure PVA powder, the second transition zone was divided into two stages between 260 and 410 °C. One part of the crystalline domain melts around 260–320 °C, which corresponds to the breaking of hydrogen bonds, resulting in a weight loss of around 25.59 wt.%. The third transition zone corresponds to additional crystalline domain melting around 320–410 °C; this is likely to be caused by the breaking of so-called strong hydrogen bonds, resulting in a total weight loss of around 51.71 wt.%. The final weight loss region peaked at 435.9 °C; this region was similar to the final region observed in the pure PVA powder, and likewise represented the carbonization process. The total weight loss that occurred in this region was only 14.51%, while the total weight loss at 500 °C was 96.32%. 

Figure 3a–c presents the thermal decomposition curve of PVAHM/70 °C. The total weight lost by PVAHM/70 °C at 500 °C was only 92.61%, which was much less than the values observed in both the pure PVA powder and PVAHM/25 °C. In the DTG curve, the temperatures of the two thermal decomposition peaks shifted further back, with the main peak appearing at 301.14 °C and the shoulder peak appearing at 352.64 °C. A similar trend was observed in the DSC pattern, with the main thermal decomposition peak and shoulder peak delayed to 313.20 °C and 357.21 °C, respectively. In addition, the small carbonization peak appeared at a higher temperature of 438.04 °C compared to the pure PVA powder (432.9 °C) and PVAHM/25 °C (435.9 °C). PVAHM/70 °C also had an extra carbonization peak of 502.18 °C. These results fully indicate that nanoscale dehydration at higher temperatures leads to more and stronger hydrogen bonds in the final PVA hydrogels. 

The crystallization behavior of the PVA material in the DSC pattern also showed that the exothermic peaks could be clearly observed in the temperature range of 200–250 °C. Compared to the PVA powder as the control, both the crystallization temperature and peak temperature of the material increased, indicating that the UND was able to increase the crystallization temperature of the PVA. This was due to the fact that the UND can increase the crystallinity of the material.

### 2.7. Fourier Transform Infrared Spectrometer (FTIR)

To understand the structure of the PVA hydrogels at the molecular scale, the infrared absorption spectra of the powdered hydrogel samples were measured using FTIR spectroscopy (Figure 3d). A very broad peak can be seen at 3447 cm^−1^ on the spectrum of the pure PVA powder (control sample); this corresponds to the stretching vibration of the hydroxyl (OH) groups. The broad peak of this stretching vibration can still be observed in PVAHM/25 °C, but its peak shifted significantly to 3394 cm^−1^, indicating that the intermolecular hydrogen bonding between the PVA chains was significantly enhanced in this sample. The intermolecular hydrogen bonding between the PVA chains of PVAHM/70 °C was also observed to be significantly enhanced, with the absorption band of the hydroxyl group stretching vibration being red-shifted from 3394 cm^−1^ to 3263 cm^−1^ and exhibiting a less broad peak. This indicates that the UND of PVAHM/70℃ resulted in stronger hydrogen bonding between the PVA main chains. The characteristic absorption peaks of the stretching vibrations of C-H and C-O appeared at 2940 cm^−1^ and 1096 cm^−1^, respectively, in the pure PVA powder [41,42]. There was no significant displacement of any of these peaks between the two PVAHMs and the pure PVA powder, except for a slight shift in the C–O peak in PVAHM/70 °C, which suggests that the oriented nanopore dehydration does not have a significant effect on these bond stretching vibrations.

### 2.8. X-Ray Diffraction (XRD) and Small-Angle X-Ray Scattering (SAXS)

The crystallinity of the PVAHMs prepared via UND was determined using wide-angle XRD (Figure 3e). Commercially available pure PVA powder was used as a control for this experiment. The PVA structure has regular regions; the extent of the sample’s crystallinity depends on the level of hydrolysis of the PVA [43]. The main peak of the pure PVA powder occurs at 19.43°, corresponding to a reflection of monoclinic crystal structure [44]. Three smaller characteristic peaks were detected at 2θ = 11.28°, 22.53°, and 40.53° [45]. It was observed that the main crystal peaks of PVAHM/25℃ and PVAHM/70℃ shifted slightly to 19.34°, although there were no significant differences in the crystal structure between the two samples. The three smaller characteristic peaks at 11.28°, 22.53°, and 40.53° were also present. These results indicate that directional dehydration did not lead to significant changes regarding the crystallinity of the PVA membranes. 

Figure 3f shows the scattering intensity–scattering vector (q) curve obtained via SAXS after correcting for the PVAHMs. Usually, the semi-crystalline structure of PVA hydrogels is manifested as microcrystalline regions dispersed into an amorphous matrix of PVA chains. The statistical average distance d between the microcrystallites can be calculated from the value of q that corresponds to the scattering peak using the equation d = 2π/q. The SAXS scattering peak becomes sharper as the size and the inter-microcrystallite distance in the system become more homogeneous. Figure 3f shows that the PVAEM prepared using water evaporation as a control sample had a small peak at 0.708 nm with an inter-crystalline spacing (d) of 8.874 nm, indicating a slight regularity in the PVA film formed by the evaporation of water from the control sample. In contrast, the intensity of the scattering peak of PVAHM/25 °C was almost 20 times greater than the control sample, and the corresponding q value had shifted to 0.598 nm, with a d value of 10.506 nm. Similarly, the intensity of the PVAHM/70 °C scattering peak increased slightly, with its q value again shifting slightly to 0.458 nm and the spacing between the microcrystalline regions calculated to be 13.718 nm. These results indicate that the UND method used in the PVAHM preparation led to larger and larger inter-microcrystallite distances and also to a smaller and smaller microcrystallites when the preparation temperature increased.

### 2.9. Scanning Electron Microscopy (SEM)

**Figure 4:**
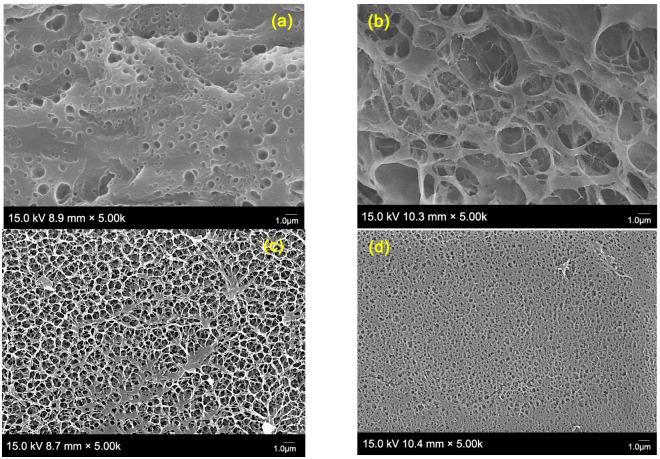
Cross-sectional SEM morphologies of (**a**) PVAEM, (**b**) PVAFT, (**c**) PVAHM/25 °C, and (**d**) PVAHM/70 °C.

To investigate the microstructural morphology of PVAHM hydrogel membranes obtained via UND, two control samples were prepared separately according to the previously reported methods [25,26,27]: a PVA cast film (PVAEM) and a film produced using five freeze–thaw cycles (PVAFT). The micromorphology of the fracture surfaces and the cross-sections of the PVA films formed using different preparation methods were observed in detail at an SEM magnification of 5000× (Figure 4). Figure 4a shows the cross-section of the PVAEM, which had a dense structure but was very thin. It had non-uniformly distributed pore sizes in the range of 0.10–1.4 μm, with a mean pore size of about 0.4 μm and a porosity of about 13.22% (Table 2). The mechanical properties of PVAEM after allowing it to swell in water were very poor (it was quite a brittle material). However, the PVAFT (Figure 4b) exhibited good mechanical properties after swelling in water, with high elasticity, a high swelling ratio, and high water absorption capacity. The porous network structure was observed at a magnification of 5000×, revealing that the pore sizes varied greatly, ranging from 0.54 to 4.29 μm, and its distribution was not uniform. The average pore size was 3.43 μm, with a porosity rate of 80.45 %. 

The thickness of the PVAHM/25 °C film was found to lie between the thicknesses of PVAEM and PVAFT. The swollen PVAHM/25 °C hydrogel was found to be soft, transparent, elastic, and possessed high mechanical strength (Figure 1d). The microstructure of the membrane cross-section was regularly arranged and had a typical porous network-like morphology with a pore size distribution ranging within 0.25–1.5 μm, an average pore size of 1.05 μm, and a porosity of 56.96%. PVAHM/70 °C exhibited a morphology and mechanical properties similar to those of PVAHM/25 °C, though it was slightly thinner. The wet film was also soft, transparent, and elastic, exhibiting a higher mechanical strength. It also revealed a typical porous network structure with smaller pore sizes ranging from 0.02 to 0.37 μm, with an average pore size of 0.27 μm and a porosity of 46.54%. These results were in accordance with the data for swelling (the equilibrium swelling ratio for PVAHM/70 °C was lower than that of PVAHM/25 °C; see Figure 2b) and indicated that the UND methodology and the dehydration temperature have significant effects on the porous network structure of PVAHM hydrogels. 

In general, the UND-based PVAHMs at 25 °C show porous network structures with an average pore size of ~1.0 μm. The PVAHMs with smaller average pore diameters can be obtained by increasing UND temperature. The PVAHM prepared in this unidirectional dehydration technology have a smaller pore diameter and a more uniform porous network structure than the hydrogel membrane prepared using the available processing methods, such as freeze–thawing and water evaporation.

### 2.10. Cell Culture

The colonization and growth of L-929 mouse fibroblasts on PVAHMs can directly reflect the biocompatibility and cytotoxicity of the biomaterial. Mouse fibroblasts were inoculated at a cell density of 10,000/mL on the surfaces of PVAHMs, as well as the control group, and the growth of the cells was observed and recorded regularly. 

**Figure 5:**
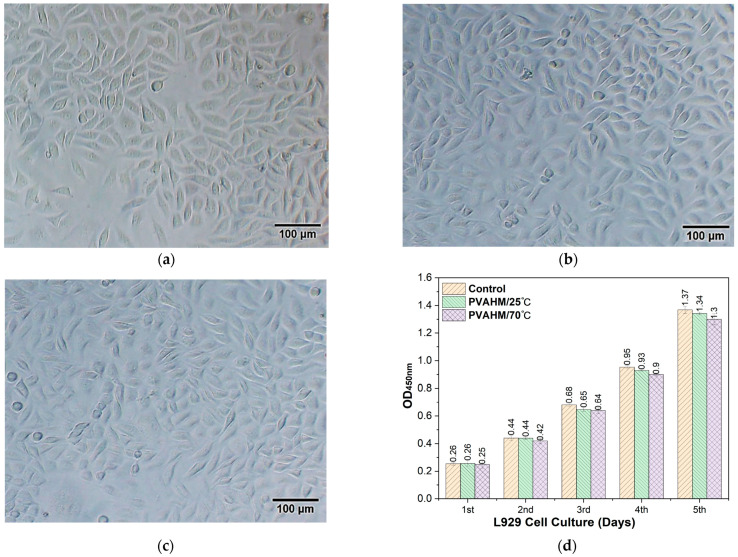
Adherence, colonization, and growth of murine L929 cells on two PVAHMs on the 5th day: (**a**) control; (**b**) PVAHM/25 °C; (**c**) PVAHM/70 °C; (**d**) cells status and growth diagram of L929 cells on PVAHM for 1~5 days (*n* = 3).

Figure 5 shows the cell status of the murine L-929 cells cultured on the two PVAHMs over five days. Compared to the control group (Figure 5a), the L-929 cells grown on the two PVAHM membranes were all adherent and normal, exhibiting either a full ellipse or shuttle shape (Figure 5b,c). There was no significant difference in the number of cells between the experimental and control groups after being cultured for 48 h. The cell viability of the PVAHMs and the control group was assessed daily for 1–5 days using a CCK-8 assay. The colonization and growth of the cells in the two PVAHMs and the control group were similar on days 1 and 2, with little change in their absorbance values measured at 450 nm. From day 3 onwards, the absorbance values at 450 nm of the CCK-8 solution as the immersion medium for the cells in the control group were greater than those for the PVAHM/25 °C group, indicating a higher cell growth rate in the control group. This trend continued on days 4 and 5, with the control group exhibiting a slightly higher cell density. While the growth rates of L-929 cells on the PVAHM/70 °C and PVAHM/25 °C samples were relatively similar, they were both slightly lower than the trends observed in the control group. The differences between the PVAHM samples and the control group on days 2–5 may have been due to the partial absorption of a water-soluble formazan produced in the presence of living cells on the porous structures of the PVAHMs, leading eventually to the lower absorbance values measured at 450 nm. These results indicate that this method of preparing PVAHMs is suitable for cell colonization and provides a good foundation for future applications as scaffolds in the field of tissue engineering, especially for implantable medical biopolymer materials. 

## 3. Discussion

In 1975, when conducting turbidimetric studies on PVA solutions, Peppas [46] used repeated freeze–thawing to prepare PVA hydrogels that exhibited hydrogen bonding at crosslinking points. Thus, the cyclic freeze–thawing method was widely used in the preparation of PVA hydrogels because of its simplicity. However, this procedure has several limitations, such as producing hydrogels with poor mechanical properties, tensile strengths, and elongation at break values that could not meet severe environmental conditions in certain applications, such as in large, unevenly sized networks and when exposed to temperatures beyond the acceptable range for biological tissues and cells during the freeze–thawing process [47]. These issues severely reduced the application of the PVA hydrogels. In the context of practical pharmaceutical and clinical applications, such as the implantation of materials into living organisms, biological hydrogels are usually required to have an anisotropic structure that not only improves their mechanical properties but that can also be used to encourage the anisotropic growth of biological tissues or organs. Unfortunately, most conventional synthetic hydrogels are essentially isotropic in terms of their microstructure and mechanical properties. Therefore, more improvements and innovations must be made to their preparation methods to develop hydrogel membranes with good cell adhesion under physiological conditions, with excellent mechanical strength, good biocompatibility, and inherent antibacterial properties; these qualities are in urgent demand in biomedical applications such as in tissue and wound healing, trauma dressings, artificial muscles, artificial cartilage, and bone tissue repair and regeneration applications.

PVA is a hydrophilic and biocompatible polymer that can be physically crosslinked into a hydrogel via cyclic freeze–thawing [25,42]. As a semi-crystalline polymer, it can form microcrystals (when its aqueous solutions are frozen at low temperatures), which can function as physical crosslinking points between the PVA chains over the entire polymer volume. After thawing, the microcrystals do not melt and instead act as strong crosslinkages that allow the PVA chains to form a three-dimensional hydrogel network. In general, the more freeze–thawing cycles used in the production of a PVA hydrogel, the slower the thawing rate, the greater the volume of crystal growth, the greater the degree of crystallinity, and the better its mechanical properties. Upon the separation of water molecules during ice formation, the hydroxyl-containing chains of the PVA are bonded by strong hydrogen interactions, resulting in a tight crosslinked layered network, which is further strengthened by van der Waals forces during repeated freeze–thawing cycles [48]. Unfortunately, PVA hydrogels prepared using conventional freeze–thawing methods are isotropic in terms of their microstructure and mechanical properties, which often results in unsatisfactory mechanical properties, with the stress and compression modulus values often being below 1 MPa [49,50].

The most common physical modification method used to address these conditions is the orientation of these hydrogels either during or after their formation. Pramanik et al. [35] synthesized PVA hydrogels of different concentrations using a liquid nitrogen directional freeze-drying technique. The microstructures of these directionally freeze-dried hydrogels exhibited lateral isotropy and a longitudinally well-aligned porous structure [32,35]. In these methods, after the hydrogel is produced by freeze–thawing, the sample is stretched to create a regular rearrangement of molecular chains [30]. Anisotropic hydrogel materials with improved mechanical properties have also been obtained via post-processing, primarily through the hot pressing of the freeze–thaw-based hydrogel [36]. Chen et al. [51] also reported the fabrication of a PVA hydrogel with an anisotropic microstructure and significantly enhanced mechanical properties. This hydrogel was prepared by first producing a freeze–thaw-based hydrogel, followed by additional cyclic freeze–thawing after elongating it by 100% or 200% in its new tensile state [51]. The stretching of the hydrogel led to the orientation of the PVA microcrystals and PVA chains, allowing the formation of more synergistic hydrogen bonds due to the freezing under stretching. The PVA hydrogels prepared using this method are much stronger than PVA hydrogels prepared via freeze–thawing alone. After only one cycle of freeze–thawing at a 100% stretching ratio, the tensile strength of such a hydrogel increased by 3.6 times compared to that of an unstretched hydrogel. Its tensile strength and elastic modulus increased significantly as the number of cycles of stretching and freeze–thawing increased. Furthermore, the elongation and amount of PVA in the direction parallel to the stretching were greater than in the perpendicular direction, meaning that the hydrogel exhibited significant mechanical anisotropy. Hydrogels prepared from aqueous 16 wt.% PVA solutions by freeze–thawing at 200% elongation exhibited tensile strength and elastic modulus values of 5.00 MPa and 0.50 MPa, respectively, in the direction parallel to the stretching [51]. However, this technique is still challenging in terms of the controllability of the process, the reproducibility of the hydrogel properties, and the bioavailability.

This study describes a new, simple, green preparation technique named UND, which allows for the preparation of PVA hydrogels with anisotropic microstructures in a porous network and significantly enhanced mechanical properties. PVAHMs with tensile strength, elongation at break, and swelling ratio values of up to ~3.0 MPa, ~350%, and ~350%, respectively, can be prepared from 10 wt.% PVA solutions at room temperature. In addition, the mechanical properties of hydrogel membranes prepared at 70℃ were even more significantly improved, exhibiting tensile strength and elongation at break values of 10.5 MPa and 891%, respectively, around 3.5 and 2 times greater than those of the PVAHMs prepared at room temperature. Furthermore, not only did the preparation at higher temperatures result in more rapid dehydration, but the average pore size of the porous network was decreased from ~1.0 μm to ~0.25 μm and the thickness of the whole hydrogel membrane was reduced from 0.55 μm to 0.45 μm (an 18% reduction). The XRD and SAXS results also showed that the use of UND during the preparation of the PVAHMs led to larger and larger inter-microcrystallite distances and also to smaller and smaller microcrystallites when the preparation temperature increased. The above-mentioned indicators of PVAHMs are intrinsically linked with the processing temperature during unidirectional dehydration through the nanopores. The temperature significantly affects the unidirectional flow rate of the water molecules through the nanopores and can alter the overall transformation of the PVA chains into physically crosslinked 3D networks. The effects of the processing temperature on the tensile strength, elongation, and elastic modulus of PVAHMs appear to reflect behavior specific to the rubber-like state. Thus, the PVA chain segments between the well-aligned microcrystalline regions in such a state undergo a micro-Brownian motion with increasing intensity due to the increasing temperature. This behavior directly results in the shrinkage of the film without the application of external stress, and on the other hand it results in increasing values of these tensile properties or the modulus of elasticity with ascending temperature. Therefore, the tensile strength, elongation, and tensile modulus of the membrane are greatly improved, but this also leads to a reduction in the pore size of the porous network in the membrane, the thinning of the membrane thickness, and an obvious reduction in the swelling ratio. At the same time, from the above results, it can be found that by adjusting the temperature during the preparation of the UND process, the above-mentioned various performance indicators for the PVAHM can be adjusted, and it can be prepared to meet various requirements for applications in actual biological materials, especially in medical biological materials. This work, thus, provides a simple, convenient, low-cost strategy for the production of anisotropic hydrogels with excellent mechanical properties, which have potential applications in biomaterials, medical tissue engineering materials, membrane separation materials, drug retardation materials, and soft mechanics.

## 4. Conclusions

UND can be used to induce the formation of PVAHMs with excellent mechanical properties from an aqueous solution of polyvinyl alcohol with a 98–99 mol% degree of alcoholysis and a molecular weight of >100 kDa. Water-stable, soft, transparent, and elastic PVAHMs were formed at 25 °C. In the wet state, the tensile strength and elongation at break were as high as ~3.0 MPa and ~400%, respectively; the tensile strength was more than 3 times higher than that of the control sample and the elongation at break was also increased by 30%. The PVAHMs prepared at 70 °C exhibited even stronger mechanical properties, exhibiting a tensile strength and elongation at break of 10.5 MPa and 891%, respectively, around 3.5 and 2 times greater than those of the PVAHMs prepared at 25 °C. The hydrogel membrane prepared at room temperature had a porous 3D network structure with an average pore size of about 1.0 μm, which was hard and firm when dry. L-929 cells were found to be able to adhere and grow well on these hydrogel membranes and exhibited high cell viability. UND can, thus, be used to induce the formation of an ordered rearrangement of PVA molecular chains to form hydrogel films with both a greater number and stronger hydrogen bonds with excellent mechanical properties. In the future, we will deeply study the exact mechanism of the formation of PVAHM using unidirectional dehydration through the nanopores and provide a theoretical basis for the extension of this method to the processing of new materials from other water-soluble polymer compounds.

## 5. Materials and Methods

### 5.1. Materials

The PVA powder was purchased from Shanghai Aladdin Reagent Co., Ltd. (Shanghai, China). Its molecular formula is [–CH_2_CHOH–]*_n_*, it has a viscosity of 54–66 mPa·s, and its molecular weight is about 190 kDa.

### 5.2. PVAHM Preparation

#### 5.2.1. Aqueous 10% PVA Solution

A certain amount of PVA powder was suspended with water, heated, and stirred at 90 °C on an electric stove until it dissolved to obtain an aqueous solution of PVA at a concentration of 10%. The beaker in which this solution was located was then sealed with kraft paper and placed in an autoclave at 120 °C for 30 min [24] to finally obtain a sterilized 10% PVA solution.

#### 5.2.2. Mold Installation and Preparation

The mold used for the preparation of the PVAHM consisted of five main parts: (1) the cup body, (2) the nanopore filter membrane, (3) a fixed ring, (4) a sampling hole, and (5) a rubber plug (Figure 1a). In this process, the bottom of the mold cup is first covered with a piece of nanopore filter membrane. The fixed flange ring is then tightened to the filter membrane at the bottom of the mold. A small amount of distilled water is added via the sampling hole using a pipette to check if the installed membrane is leaking; if so, the membrane must be reinstalled until no leakage occurs. After the sample solution is added, the sampling hole is sealed with a rubber stopper. The mold is then placed on a mold holder with the membrane facing downward, and a miniature fan can be placed underneath the membrane to speed up the airflow and accelerate the nanopore dehydration. The size of the pores in the nanopore membranes is generally ≤1000 kDa of the molecular weight cut-off or ≤50 nm. 

#### 5.2.3. PVAHM Preparation

As shown in Figure 1a, 5.0 mL of aqueous 10% PVA solution was added to the mold using a pipette. The chamber was sealed by plugging the sampling hole with a rubber plug. The mold was placed on a horizontal mold stand at 5–50 °C and at 10–90% relative humidity (RH) for unidirectional dehydration. After about 5–16 h, the PVA aqueous solution in the mold cup will have dehydrated, forming a flakey, water-insoluble PVA film on the nanopore filter membrane. After it is immersed in water, a soft, elastic, and transparent PVAHM is obtained by removing it from the filter membrane. 

### 5.3. Analytical Methods

#### 5.3.1. Mechanical Properties

Prior to the measurement of its mechanical properties, the dry PVA hydrogel film was immersed in deionized water at 25 °C for 24 h. After removing the excess water on the surface, the film in a swollen state was then cut into long strips of 30 mm × 5 mm × 0.5 mm. Then, the strips were placed on the tensile instrument. The data were recorded and the mean and standard deviation for each sample were calculated. The tensile strength was obtained according to the following formula.

σ (MPa) = T (N)/S (mm^2^) (1)

Here, σ is the tensile strength, T is the tensile stress, and S is the cross-sectional area.

#### 5.3.2. Swelling Ratio Test

The degree of swelling is related to the ability of biological scaffolds to absorb water, which is an important evaluation index. The swelling ratios of the prepared PVA hydrogel membranes considered as biological scaffolds in each group were calculated as follows: (1) the groups of biological scaffolds were placed in a desiccator, dried overnight at 37 °C, and weighed (recorded as M_0_); (2) the dried biological scaffolds were immersed in centrifuge tubes containing excess deionized water and placed in a biochemical incubator at 37 °C to allow them to absorb water and swell; (3) the scaffolds were removed every hour and filter paper was used to wipe off any surface water on the sample, then the sample was weighed again (recorded as M_n_); (4) the sample swelling ratio values were derived from Equation (2). These measurements were performed in triplicate:

Swelling ratio (%) = (M_n_/M_0_) × 100% (2)

#### 5.3.3. Scanning Electron Microscopy Investigation

The groups of PVAHM samples that reached their swelling equilibrium were frozen in liquid nitrogen. The membranes obtained in this way were cracked using tweezers and lyophilized at −80 °C. Then, the surfaces of the thin flaky scaffolds were sprayed with gold for 70 s; this allowed the surfaces of the samples as well as the apparent cross-sectional morphology to be better observed under a scanning electron microscope (SEM; Regulus 8230, Hitachi, Tokyo, Japan). An accelerating voltage of 15 kV was used for all images acquired. 

#### 5.3.4. Thermal Analysis

To compare the thermal properties of PVAHMs produced using different preparation methods, the PVAHMs were first ground into powders. Approximately 8.0 mg of each sample was weighed out and a TGA was conducted using a differential thermogravimetric coupler (SDT2960, TA, DE, USA). The data were processed via derivatization and the TG, DTG, and DSC curves were plotted. The test parameters were as follows: nitrogen protection, with a temperature range from 25–900 °C and a ramp rate of 10 °C/min. 

#### 5.3.5. FTIR Spectroscopy Analysis

The PVAHMs samples were milled into powders; the PVAHM films were also directly analyzed on a Fourier transform infrared spectrometer (FTIR; Nicolet 6700, Thermo Fisher, Waltham, MA, USA). Sixteen scans were performed with a resolution of 4 cm^−1^ in a spectral range of 2000–1000 cm^−1^.

#### 5.3.6. Powder X-Ray Diffraction Study

The prepared PVA hydrogel films were air-dried and ground into powder. The sample powders were analyzed using an X-ray diffractometer (D8 Advance, Bruker, Karlsruhe, Germany). The operating XRD parameters were as follows: a copper target, with a tube voltage of 40 kV, tube current of 40 mA, X-ray wavelength of 0.15 nm, range of diffraction angles (2θ) of 5–50°, scanning step size of 0.03°/s, and scanning speed of 12.5°/min.

#### 5.3.7. Small-Angle X-Ray Scattering (SAXS)

To investigate the ordered structure of the PVAHMs, a small-angle X-ray scattering system (SAXS; Anton Paar SAXSess mc^2^, Graz, Austria; wavelength of 0.1542 nm, copper target, 40 kV, 50 mA) was employed. The film samples were installed on the membrane cell of the multifunctional sample stage. A blank cell was used to determine the background scattering. The sample and background scattering were measured for a certain period of time, and these measurements were used to normalize the intensity of the scattered beam.

### 5.4. Cell Culture

#### 5.4.1. Cell Culture Procedure

L-929 mouse fibroblasts were selected for the cytocompatibility assay to determine the cell viability of these cells grown on the hydrogel scaffolds [52]. The culture medium was Dulbecco’s modified Eagle’s medium (DMEM) containing 10% fetal bovine serum and 1% penicillin. The parameters of the cell culture incubator were set to a temperature of 37 °C and a CO_2_ concentration of 5%. The L-929 cells were removed from the liquid nitrogen, immersed in a water bath at 37 °C, supplemented with 4 mL of medium, and centrifuged (800 rpm, 5 min). The supernatant was then discarded and 2 mL of medium was added to the cellular sediment. The suspension was transferred to two cell culture flasks, supplemented to a volume of 5 mL with the medium, and placed in the incubator. The L-929 cells were allowed to grow to 80–90% before the cell passages were performed. The cells were washed with PBS, 1 mL of 0.25% trypsin was added to the cells, and they were digested at 37 °C for 1 min. When the cells retracted and became round, 4 mL of the culture medium was added to terminate the digestion. The cells were blown down gently and centrifuged (800 rpm, 5 min). Once again, the supernatant was discarded, 2 mL of the medium was added, and the suspension was transferred to two cell culture flasks, made up to a volume of 5 mL using the culture medium, then the culture was allowed to continue. Finally, cell counting was performed by means of an inverted microscope after diluting the cell suspension to a suitable concentration. 

#### 5.4.2. Cell Pavement

The PVA membranes were prepared in the form of 1.0-cm-diameter circular sheet-like scaffolds using a punch machine and placed into a 48-well plate. Then, 500 µL of 75% ethanol was added dropwise to each well and aspirated after 10 min. A sufficient amount of PBS was added to each well and allowed to soak for 10 min—this process was repeated twice. Finally, 500 µL of the complete medium was added to each well, submerging the biological scaffold. The samples were set aside and left overnight. The medium was then discarded and the L-929 cells to be passaged were counted as described in the previous section. Next, 500 µL of the cell suspension was added at a density of 104/mL to each well; the cells were then incubated. 

#### 5.4.3. Cell Morphology Observations

During the incubation period, the 48-well plates were removed from the incubator each day and observed under an inverted fluorescent microscope to record the cell growth. The cells were fluorescently stained using a stock solution of calcein AM (for living cells) and propidium iodide (PI) reagent (for dead cells). The solution was equilibrated at room temperature for 30 min. Next, 2 µL of 16 mM PI stock solution and 4 mL of PBS were mixed together to obtain an 8 µM PI solution. Then, 2 µL of 4 mM calcein AM stock solution was added to this mixture to result in a working solution of 2 µM calcein AM and 8 µM PI. Next, 500 µL of PBS was added to each well to wash the cells, and afterwards they were aspirated and the process was repeated once more to remove the active esterase within the medium. Finally, 100 mL of the working solution was added dropwise to each well while making sure that the cells were not covered. The cells were incubated for 30–40 min at room temperature and fluorescence micrographs were taken using an inverted fluorescence microscope.

#### 5.4.4. Cell Viability Assay

To investigate the effects of PVAHM scaffolds on the viability of L-929 cells, CCK-8 was used to determine daily the cell viability in the presence of various biological scaffolds. A 48-well plate was spiked with 10 µL of CCK-8 solution per well and incubated for 3 h. To reduce experimental errors and avoid the effect of the bioscaffold on the OD values, the cell cultures to be tested in each group were transferred to a new 48-well plate and the OD values of each well were measured at 450 nm. This process was repeated five times for each group.

### 5.5. Data Processing

The data were analyzed using Origin 9.1 software (version 91E). The results are reported as means ± SD. An ANOVA test was used to evaluate the statistically significant differences between groups. 

## Figures and Tables

**Table 1:** Effect of the PVA type on the formation of UND-based PVAHMs and their properties.

Mowiol^®^	Alcoholysis Degree (%)	Mw (kDa)	Water Stability	σ (MPa)	±SD	EB (%)	±SD	E (MPa)	±SD	Note
PVA-105	98~99	~47	√	0	0	0	0	0	0	
PVA-117	98~99	~145	√	1.19	0.25	333	73	0.43	0.07	toxicity
PVA-124	98~99	~190	√	2.57	0.46	409	36	0.59	0.02	in use
PVA-203	87~89	~31	×	—	—	—	—	—	—	—
PVA-210	87~89	~67	×	—	—	—	—	—	—	—
PVA-224	87~89	~205	×	—	—	—	—	—	—	—

Note: √, PVAHM made by UND method can maintain a stable membrane form in water and will not dissolve; ×, PVAHM made using UND method that is soluble in water; —, there is no way to determine the mechanical properties of the PVAHM in the wet state because it is soluble in water. Mw means the molecular weight, σ stands for the tensile strength, EB stands for the elongation at break, and E stands for the modulus of elasticity.

**Table 2:** Statistics data on the pore size and pore size distribution based on SEM data.

No.	Hydrogels	Mean Size (µm)	±SD	Max	Mini	Pore Number	±SD	Porosity (%)	±SD
a	PVAEM	0.40	0.30	1.40	0.10	664	3.20	13.22	5.51
b	PVAFT	3.43	1.05	4.29	0.54	180	5.03	80.45	10.94
c	PVAHM/25 °C	1.05	0.27	1.50	0.25	1263	1.72	56.96	0.71
d	PVAHM/70 °C	0.27	0.08	0.37	0.02	1484	1.03	46.54	1.30

## Data Availability

The datasets used and/or analyzed during the current study as well as the analysis scripts are available from the corresponding author upon reasonable request.

## Abbreviations

UND	unidirectional nanopore dehydration
PBS	phosphate-buffered saline
TG	thermogravimetric
DTG	differential thermogravimetric
DSC	differential scanning calorimetry
DMEM	Dulbecco’s modified Eagle’s medium
calcein AM	calcein acetoxymethyl ester
OD	optical density
PVAHM	PVA hydrogel membrane
